# Incidence and Determinants of Spontaneous Cardioversion of Early Onset Symptomatic Atrial Fibrillation

**DOI:** 10.3390/medicina58111513

**Published:** 2022-10-24

**Authors:** Marco Valerio Mariani, Nicola Pierucci, Agostino Piro, Sara Trivigno, Cristina Chimenti, Gioacchino Galardo, Fabio Miraldi, Carmine Dario Vizza

**Affiliations:** 1 Department of Clinical Internal, Anesthesiologic and Cardiovascular Sciences, Sapienza University of Rome, Viale del Policlinico 155, 00161 Rome, Italy; 2 Medical Emergency Unit, Sapienza University of Rome, Viale del Policlinico 155, 00161 Rome, Italy

**Keywords:** atrial fibrillation, spontaneous cardioversion, antiarrhythmic therapy

## Abstract

Atrial fibrillation (AF) is the most frequent chronic arrhythmia worldwide, and it is associated with significant morbidity and mortality, making it a considerable burden both to patients and the healthcare system. Nowadays, an early attempt to restore sinus rhythm in acute symptomatic AF through electrical or pharmacological cardioversion is the most common approach in the Emergency Department (ED). However, considering the high percentage of spontaneous cardioversion of paroxysmal AF reported by many studies, this approach may not be the ideal choice for all patients. In this manuscript we performed a review of the most relevant studies found in literature with the aim of identifying the main determinants of spontaneous cardioversion, focusing on those easy to detect in the ED. We have found that the most relevant predictors of spontaneous cardioversion are the absence of Heart Failure (HF), a small atrial size, recent-onset AF, rapid Atrial Fibrillatory Rate and the relationship between a previous AF episode and Heart Rate/Blood Pressure. A number of those are utilized, along with other easily determined parameters, in the recently developed “ReSinus” score which predicts the likelihood of AF spontaneous cardioversion. Such identification may help the physician decide whether immediate cardioversion is necessary, or whether to adopt a “watch-and-wait” strategy in the presence of spontaneous cardioversion determinants.

## 1. Introduction

Atrial fibrillation (AF) is the most frequent chronic arrhythmia worldwide [1,2,3,4,5,6], and it is associated with substantial morbidity [7] and mortality [8], making it a significant burden to patients and the healthcare system. Progressive aging of the population and increase in the prevalence of AF-predisposing risk factors [9], such as systemic arterial hypertension [10,11,12,13,14,15], coronary artery disease (CAD) [16,17,18,19], HF [20,21,22,23,24], including HF with preserved ejection fraction [25,26] (HFpEF), chronic kidney disease (CKD) [27,28,29,30,31,32], Obesity [33,34], Obstructive Sleep Apnea [35], but also psychological problem such as anxiety [36] are predictors of a future increase both in the incidence and prevalence of AF and AF-related healthcare costs [37].

The currently estimated prevalence of AF in adults is between 2% and 4%, and a 2.3-fold rise is expected, attributable to the extended longevity of the population. It has been estimated that this condition will affect 6–12 million people in the US by 2050 and 17.9 million people in Europe by 2060 [38]. As a consequence of its relative frequency in the adult population it represents one of the main cardiovascular causes of ED visits [39].

Studies have estimated that in 2005 the annual AF-related costs reached $6.65 billion in the United States and £459 million in the United Kingdom [39,40], representing a considerable proportion of the total healthcare economic burden [41,42]. Direct costs derive from the management of AF in the ED, hospitalizations, complications such as HF, and the need for anticoagulation therapy which requires drugs, follow-up visits and periodic INR testing [43]. Indirect costs of AF are mainly related to the consequences of disability caused by stroke and the substantial productivity loss in younger cohorts [44].

Nowadays [45], an early attempt to restore sinus rhythm (SR) of acute symptomatic AF through electrical cardioversion (ECV) or pharmacological cardioversion (PCV) [46,47,48,49,50,51] is the most common approach in the ED [52,53,54,55]. ECV is a relatively expensive procedure [56,57], requiring the involvement of nurses, an anesthesiologist and a cardiologist or emergency physician, whereas PCV may be time-consuming, especially when attempted with the use of amiodarone infusion, resulting in long observation times in the ED or during hospitalization. However, several studies have reported the results of a “wait-and-see” approach, with spontaneous conversion (SCV) of AF to SR occurring frequently in the ED, with rates as high as 70%. Therefore, an appropriate identification of factors associated with spontaneous cardioversion of recent-onset AF may help clinicians select patients suitable for a wait-and-see approach and early ED discharge rather than PCV and ECV procedures, reducing healthcare costs and length of hospital stays. The aim of this review is to identify the characteristics associated with SCV in patients presenting with hemodynamically stable AF in the ED.

The ED represents the setting where AF is most often diagnosed and treated [58]. The main priority following first diagnosis of AF is to provide adequate anticoagulation. Anticoagulation, preferably with DOACs [59,60], is the cornerstone of AF treatment as it helps to prevent adverse thromboembolic events such as ictus [61]; this also holds true for fragile subjects [62], including patients with chronic renal insufficiency [63,64,65,66]. Once anticoagulation is guaranteed, current ESC guidelines and real-world practice aim to treat AF as soon as possible to achieve SR. The primary indication for rhythm control is to reduce AF-related symptoms [67,68,69] and improve quality of life (QoL) [70,71,72]. AF progression is associated with a deterioration of heart function [73,74,75] and QoL and if not treated in a timely manner, it becomes harder to treat or irreversible [76]. Therefore, it may be appropriate to consider rhythm control as soon as possible in hemodynamically stable patients with symptomatic AF. Regarding the treatment method, in hemodynamically stable patients, either pharmacological cardioversion or electrical cardioversion can be used to achieve SR. However, deciding if immediate action for restoration of sinus rhythm is necessary, or whether spontaneous conversion can be expected, remains challenging, as no objective decision-making guidance is currently available.

The most important aspect to consider when choosing the best therapeutic approach for AF is time of onset. If AF onset was less than, or up to 48 h before diagnosis [77,78], cardioversion can be attempted without relevant risk of stroke [79]. The latest ESC guidelines suggest that ideal candidates for immediate CV have no previous thromboembolic events and a CHA2DS2-VASc [80,81,82] score of ≤1 in males and ≤2 in females [83,84]. On the contrary, if AF is presumed to have started more than 48 h before diagnosis, it is necessary to initiate anticoagulation therapy at therapeutic doses for at least 3 weeks, or perform a transesophageal echocardiogram in order to exclude thrombi in the left atrial appendage before attempting cardioversion. The best candidates for delayed CV have a previous history of thromboembolism, a mechanical prosthetic heart valve [85,86], mild or severe mitral stenosis and a CHA2DS2-VASc score of ≥2 in males and ≥3 in females.

In the case of a hemodynamically unstable patient, immediate cardioversion is recommended regardless of the time of onset; synchronized direct electrical cardioversion [87] is the best choice in hemodynamically compromised AF patients as it is more effective than pharmacological cardioversion and results in immediate restoration of sinus rhythm.

Pharmacological cardioversion to sinus rhythm is an elective procedure indicated for hemodynamically stable patients; its true efficacy is biased by the common spontaneous restoration of sinus rhythm within 48 h of hospitalization as documented by several studies. Therefore, a ‘watch-and-wait’ strategy (usually lasting <24 h) is also suggested in the latest ESC AF Guidelines, and it may be considered in selected, hemodynamically stable patients with recent-onset AF as a non-inferior alternative to early cardioversion, taking into account the high rate of SCV described by several studies. The current challenge is the identification of those characteristics that will favor spontaneous cardioversion in order to reserve electrical or pharmacological cardioversion to those patients who won’t spontaneously reverse back to SR.

## 2. Materials & Methods

A comprehensive search on the NCBI Platform was performed by 2 reviewers (M.V.M. and N.P.) for the initial study inclusion. Keyword used for the search were the ones indicated in this manuscript: AF, antiarrhythmic therapy and spontaneous cardioversion. No restrictions on study setting, year, place, or language were set on the first screening. Studies identified during title or abstract screening were included for full-text review. A second round of eligibility check was performed after retrieval of the full text. A total of 10 studies, both prospective and retrospective, were found to be eligible for inclusion in this review. 

## 3. Results

### 3.1. Main Determinants of AF Spontaneous Cardioversion

It is commonly reported that AF may convert spontaneously into SR without medical intervention: several studies show various determinants of potential spontaneous cardioversion into SR; recognizing them could help tailor the therapeutic approach to each individual patient: a “watch-and -wait” strategy for those who will likely cardiovert spontaneously, or an interventional approach for those who lack spontaneous cardioversion factors.

### 3.2. Heart Failure

First evidence of spontaneous cardioversion derived from the study of Galve et al. [88] in 1996, which compared the efficacy of intravenous amiodarone (5 mg/kg in 30 min followed by 1200 mg over 24 h) vs placebo in the treatment of recent onset AF (<1 week); in this study no difference between these two approaches emerged in the 24 h of follow up in the ED (*p* = 0.532). By the end of the 24-h treatment period, 34 patients (68%) in the amiodarone group and 30 patients (30%) in the control group had reverted to sinus rhythm, respectively (*p* = 0.532). From a multiple logistic regression analysis, the absence of congestive HF was considered an independent predictor of spontaneous conversion to sinus rhythm. The confirmation of the influential role of HF came from the study by Boriani et al. (1997) [89] which evaluated the efficacy and safety of Propafenone vs placebo in restoring SR in recent onset AF (<1 week); Propafenone was more effective than placebo in converting AF to sinus rhythm at 3 h (*p* < 0.001) and 8 h (*p* < 0.02). Subgroup analysis showed that among patients without heart disease, 78% of those receiving propafenone and 56% of those receiving placebo converted to sinus rhythm within 8 h (*p* = 0.02); in patients with structural heart disease, the rate was 81 % for those receiving propafenone and 17% for those receiving placebo (*p* < 0.001). Spontaneous cardioversion rates for patients receiving placebo differed significantly: 56% for patients without structural heart disease, 27% for patients with hypertension, and 17% patients with structural heart disease (*p* = 0.009). For patients who did not present with HF the probability of conversion to sinus rhythm at 3 h was higher in the propafenone group than in the placebo group (48% for propafenone compared with 26% for placebo; *p* = 0.02). The interventional approach was deemed necessary for patients with heart disease; rates of spontaneous conversion to sinus rhythm after the onset of AF among such patients was extremely low. Of note, patients with NYHA > II were unfortunately excluded from this study. Another paper confirming the leading role of the absence of HF in SCV was the one from Cotter et al. (1999) [90] comparing the effect of high-dose intravenous amiodarone (3 g in 24 h) vs Placebo in the treatment of acute AF, in patients who had had a previous AF episode, and were not under anti-arrhythmic drug (AAD) treatment. The conversion rate in the 24 h of treatment in the amiodarone group was 92% (46/50, *p* = 0.0017 compared to the placebo group), while conversion to normal sinus rhythm within 24 h occurred in 32 out of 50 patients (64%) in the placebo group, most of whom converted within 8 h. Lower conversion rates were observed in patients with hypertension, ischemic heart disease or congestive HF and in patients with echocardiographic findings such as ejection fraction below 45% or significant mitral regurgitation. Most importantly, it appeared that in patients with favorable echocardiographic findings the rate of spontaneous conversion was approximately 90% in the first 24 h. Although intravenous high-dose amiodarone (3 g in 24 h) safely facilitated conversion of recent-onset paroxysmal AF, the high rates of spontaneous conversion observed suggest that such treatment should be reserved for patients with unfavorable risk factor profiles, not converting within 8 h of observation or requiring rate control. The study of Lindberg et al. (2012) [91] contributed to the definition of HF as one of the main determinants of SCV, studying 374 patients admitted to the ED for first episode of AF. They investigated the rate of SCV and its strongest determinants: slightly (45–50%) and severely (<45%) reduced LVEF decreased the probability of spontaneous conversion (OR = 0.3; *p* = 0.004), whereas with normal LVEF there was a tendency for spontaneous conversion. However, this was not found to be statistically significant (OR = 1.4; *p* = 0.2).

### 3.3. Left Atrial Size

The main contribution to the relevance of left atrial dimensions as a predictor of SCV comes from a study by Geleris et al. (2001) [92] whose purpose was to determine the likelihood of spontaneous conversion of recent onset (<24 h), paroxysmal AF to sinus rhythm based on clinical and echocardiographic predictors, during a monitoring period of at least 24 h. Notably, hemodynamically unstable patients and those who presented with recent myocardial infarction, unstable angina, average ventricular rate > 150 beats/min, hyperthyroidism, congestive HF, left ventricular hypertrophy, valvular heart disease, or on antiarrhythmic drugs at the time of admission were excluded from this study. Spontaneous conversion to sinus rhythm occurred in 109 patients (71.2%); among those, 73.4% converted in the first 12 h. Left atrial diameter was significantly greater in patients who achieved SCV compared to those who did not achieve SCV (*p* < 0.03); likewise increased left atrial diameter (>40 mm) was more often seen in patients without SCV compared to patients with spontaneous conversion (45% vs. 22%, *p* < 0.05). Similar evidence about the important influence of left atrial dimensions on SCV comes from two studies already mentioned in this manuscript, namely Galve et al. (1996) [88] and Cotter et al. (1999) [90]. In the paper by Galve et al. multiple logistic regression analysis showed that a smaller left atrial size (Diameter 41 ± 7 mm) was an independent predictor of conversion to sinus rhythm (*p* = 0.0095). According to Cotter et al. [90] a left atrial size below 45 mm (along with FE > 45% and no significant mitral regurgitation) guarantees a spontaneous cardioversion rate of 90% (*p* = 0.003).

### 3.4. Duration of AF

Many studies agree that AF of shorter duration, increases the likelihood of spontaneous cardioversion to SR. The study by Danias et al. (1998) [93] aimed to evaluate the predictors and likelihood of spontaneous restoration of sinus rhythm in recent-onset AF (symptoms lasting < 72 h). Patients on AADs were excluded from the study. Spontaneous conversion to sinus rhythm occurred in 68% of the study group (*n* = 242/356). Among patients achieving spontaneous conversion, the total AF duration was <24 h in 159 (66%), 24 to 48 h in 42 (17%) and >48 h in 41 (17%) (*p* < 0.001). Logistic regression analysis of clinical data identified presentation to the ED within <24 h from symptom onset as the only predictor of spontaneous conversion (*p* < 0.0001).

In 1999, Dell’Orfano et al. [94] performed a retrospective medical chart review of all patients who presented to the ED with a primary diagnosis of AF; patients were excluded if they had received a different primary diagnosis and had secondary AF, if they were only treated in the ED and subsequently discharged without hospital admission, and patients admitted for AF who sought initial treatment in a non-ED setting. Out of 114 patients, 88 (77%) successfully converted to sinus rhythm; of those 88, 57 (65%) spontaneously converted. It was observed that those with AF duration <48 h were more likely to convert (*p* = 0.0001). Spontaneous reversion to sinus rhythm happened in the first hour in 14 patients (25%), in 1 to 12 h in 24 patients (42%), in 12 to 24 h in 16 patients (28%), and in 24 to 48 h in 3 patients (5%). Length of hospital stay for those who spontaneously converted was 2.4 ± 1.7 days, which is significantly shorter than the 4.7 ± 2.9 days for non-converters (*p* = 0.0001). Regarding costs, the hospital charges for spontaneous converters were lower, reaching $4930 ± $2741 versus $9892 ± $6703 for those who underwent electrical cardioversion (*p* = 0.0001), while they came up to $6909 ± $3358 for patients who did not convert (*p* = 0.002). These data suggest that the acute treatment of AF is not only costly, but also associated with a longer length of hospital stay. The mean length of stay was 3.9 ± 5.2 days and the hospital charges averaged $6692 ± $4928. Therefore, this study showed that patients who spontaneously cardioverted had lower hospital charges and shorter lengths of stay, suggesting that in selected patients heart rate control and brief observation may be a good and cost-effective treatment strategy. In the acute hospital setting, non-tailored treatment of AF can be quite costly and is associated with a longer length of stay. Further confirmation of the importance of AF duration comes from the study by Lindberg et al. (2012) [91] who, retrospectively reviewed charts of 438 consecutive patients admitted to the hospital with first-onset AF with the aim to determine the probability of spontaneous conversion to sinus rhythm and to identify predictive factors of conversion. In this study, patients were divided in two groups: those with AF lasting < 48 h and AF lasting > 48 h. Patient were excluded from the study if they had a previous episode of AF, or if duration of AF was <5 min. Conversion was defined as spontaneous if it occurred before 48 h from admission and the patient did not receive active cardioversion (electrical cardioversion or via administration of anti-arrhythmic drugs). Spontaneous conversion to SR occurred in 54% (*n* = 203/438). In the group with first onset AF < 48 h, spontaneous conversion occurred in 77%, compared to 36% in the group with first onset AF > 48 h. Logistic regression analysis identified duration of AF as a highly significant predictor of spontaneous conversion to sinus rhythm (*p* < 0.001). Remarkably, no thromboembolic complications after spontaneous conversion were observed during admission. Therefore, as no complications to spontaneous conversion were observed, those cases could potentially be managed as outpatients.

### 3.5. Atrial Fibrillatory Rate

Choudary et al. (2013) [95] contributed to our understanding of the main causes of SCV. In previous studies, Atrial Fibrillatory Rate (AFR) has been linked to the duration of atrial refractory periods and is considered a non-invasive index of atrial remodeling. A great amount of studies have proved that AFR is also a predictor of outcome in patients with persistent AF, finding an association between good outcomes of catheter ablation and cardioversion and low AFR. This is the first study which investigated AFR in a non-selected population sample represented by 148 patients with AF episodes of short duration.

The aim of the study was of predict the rate of SCV in patients with recent onset AF (lasting < 48 h). Over a period of 12 months, a total of 148 12-lead ECGs at ED admission were extracted from the hospital database to be processed offline. In order to isolate and analyze the atrial component of the ECG, all ECGs were first pre-processed. That consisted in cross-correlation-based beat classification and, beat identification as well as baseline filtering. Subsequently, spatiotemporal cancellation of the QRST complexes was done. This was achieved through the subtraction of the average QRST from each beat in the ECG, calculated according to morphology and amplitude. At the end of this process of ECG filtering, the resulting ECG mainly contained the atrial activity. This remaining atrial activity on ECG was examined by using characterization of sequential atrial signal [96]. This technique allows a time-frequency analysis by overlapping short duration windows in order to provide AFR trends second-by-second. The rate of AF was then obtained thanks to an AFR software (Cardiolund Research AB) [97] that only analyzes lead V1 for a total of 10 s on a digital 12 lead ECG [98].

Among the 148 patients, AFR at the moment of admission was 352 + 57 fibrillations/minute (fpm) (median 350 fpm, range 191–490 fpm). Using Kaplan–Meier analysis, patients with an AFR under the median of this study population (AFR = 350 fpm) were found to more frequently experience spontaneous restoration to SR in comparison with those with higher AFR (*p* = 0.017). The main predictors of early SCV were assessed in a subgroup of patients who did not receive any cardioversion attempt within the first 18 h of symptom onset (*n* = 105, AFR 351 + 59 fpm). The 18-h cutoff was chosen retrospectively to obtain equal size patient groups, and to set a time range with clinical relevance for predicting spontaneous conversion. Patients who had a spontaneous conversion to SR within 18 h had an higher prevalence of first-ever episode of AF and the majority was female, if compared to patients who had not a spontaneous conversion to SR within the first 18 h. Through the multivariate analysis, two parameters have been proved to be independent predictors of SCV within 18 h: a first-ever episode of AF and an AFR up to 350 fpm. Moreover it was found a negative correlation between AFR and age (*p* = 0.001): in fact those with an age over the population median (age > 65 years) were characterized by a lower mean AFR of 329 ± 50 fpm, as compared to those younger than 65 years (376 ± 55 fpm). Female patients had a lower AFR than male patients (333 ± 53 vs. 367 ± 56 fpm, respectively, *p* = 0.001). There was no correlation between AFR and left atrial (LA) diameter. Furthermore, AFR was significantly higher in patients with a first-ever episode of AF (376 + 64 vs. 345 + 53 fpm, *p* = 0.012). A possible explanation for the higher AFR in first-ever episodes of AF could be the lack of atrial structural remodeling, (i.e., atrial fibrosis), which would have determined the slowing of the atrial fibrillatory process. In that population, the majority (85%) of patients experiencing their first-ever AF episode had absence of structural heart disease, defining the so-called ‘lone AF’, which supports the aforementioned explanation. Another possible explanation could be that the rapidly firing foci within the pulmonary veins function as a trigger of lone AF, which may result in a higher fibrillatory rate.

The AFR value of 350 fpm, along with a first-ever episode of AF adds significant information for identifying who would most likely convert spontaneously within 18 h, suggesting that electrical cardioversion in patients with a high likelihood of SCV can be postponed while priority for cardioversion should be given to those who are unlikely to convert spontaneously. This finding has been explained by electrophysiological changes of ageing atria, and structural atrial abnormalities found in the eighth decade of life. However, Choudary et al. [95] has recently reported that the extent of structural changes in the atrial wall was not associated with age, but was instead correlated with AF presence and persistency of AF, suggesting that age-related structural alterations are not the onlyparameter determining AFR.

### 3.6. The Relationship between Heart Rate, Blood Pressure and the Number of Past Episodes of AF

An original contribution to the quest for the definition of the most relevant determinants of SCV comes from the study of Perrea et al. (2011) [98] who created a formula which contains Heart Rate, Blood Pressure and the number of past episodes of AF to predict the probability of SCV. This is a retrospective study which tried to elaborate a formula that would have predicted who, out of the patients that presented to the ED with paroxysmal AF (<48 h from the beginning of symptoms), would achieve SCV and who would respond to the initial i.v. amiodarone dose.

Spontaneous SR restoration prognostic parameters were identified using logistic regression analysis: what emerged from the regression model was that the most significant predictor was heart rate at admission. Moreover, the most important predictors of 24-h hospitalization were determined to be heart rate at admission as well as the number of AF episodes experienced in the past. In the second step of the analysis, the derived ratios were examined to check for statistical significance, the only parameter achieving it being the heart rate/systolic BP ratio. The binary logistic regression model including the heart rate/systolic BP ratio and the number of past AF episodes was the one which had the highest predictive accuracy. Therefore, by incorporating the number of past AF episodes, the formula [(heart rate/systolic blood pressure) × 0.1 × number of past AF episodes], achieved sensitivity 78.6% and specificity 77.9% (*p* = 0.001) with a cut-off of 1.3. This formula was finally chosen as the one that combined the highest predictive accuracy and ease of use. The cutoff of 1.31 was derived from binary logistic regression: above that value SCV is very likely, below that it’s less probable. As an example, a patient with a heart rate of 125 beats/min, systolic BP of 162 mmHg, and one past AF episode ([125/162] × 0.1 × 1) will have a formula result of 0.87. On the contrary, a patient presenting with a heart rate of 130 beats/min, a systolic BP of 112 mmHg, with a previous history of two AF episodes, has an equation result of 1.36, which is greater than the cutoff of 1.31 and, therefore, this patient would be more likely to convert to SR. This formula can be easily and rapidly calculated using parameters defined at the time of admission, has high sensitivity and moderate specificity, does not demand financial or human resources—so it can be considered cost effective—and is easily applied in the ED setting with the possible result of shortened time of hospitalization and healthcare-related costs.

### 3.7. Biological Clockwork and Hormonal Factors

Mattioli et al. (2000) [99] discovered two relevant factors for SCV: in this prospective study 140 consecutive patients with a mean age of 60 ± 13 years presenting in the ED with symptomatic lone AF were enrolled and observed for 48 h; inclusion criteria were AF onset of less than 6 h before ED presentation. Out of a population of 140 patients, 108 (77%) patients underwent SCV within 48 h, while the other 32 received active cardioversion, either electrical or pharmacological.

By Logistic regression analyses of echocardiographic, clinical and neurohumoral data emerged that the most important predictor of spontaneous conversion was when, during the day, did AF have its onset: patients who developed the arrhythmia during sleep had the highest probability of spontaneous conversion during the first 24 h (OR 6.7; 95% CI 5.9 to 9.5). Another important aspect is that the plasma concentration of Atrial Natriuretic Peptide (ANP) >300 ng/L during the arrhythmia was the second predictor of spontaneous conversion (OR 3.24; 95% CI 2.30 to 6.46): the higher the value of ANP during the AF episode, the higher the probability of SCV. This correlation could be explained by the fact that the levels of ANP reflect the activity of atrial structures: Van den Berg et al. [100] found that the ANP level was lower in patients with AF of longer duration. The investigators attributed this finding to a failure of the atria to produce ANP because of degenerative changes caused by AF: this arrythmia, if chronic, provokes variations in the histologic features, size, and endocrine function of the atrium. Recent onset AF is presumed to not have had the time to reduce the activity of atrial tissue, and consequently atria with a normal endocrine function are more likely to undergo SCV. So, ANP is a double-faced hormone: in recent onset AF a high level (>300 ng/L) predicts SCV while in chronic AF the impaired AF function is correlated with low levels of ANP. Notably, in this study age, gender, left atrial dimension and duration of AF weren’t found to be statistically relevant predictors for SCV.

### 3.8. Recent Findings

The newest observational study regarding this topic comes from Pluymaekers et al. (2021) [101]; this study confirms and expands the results found in previous studies, shedding light on the management of new-onset AF in the ED. The originality of this study consists in shortening the observation time for SCV at 3 h from ED access. This time interval could be useful in clinical practice for deciding who should receive cardioversion, either electrical or pharmacological, and who may undergo SCV. Out of 943 consecutive patients—by far the largest study population—with a mean age of 69 ± 12 years, spontaneous conversion to SR occurred in 158 patients (16.8%). The number of patients in SR after 1 h was 100 (10.6%), and after 2 h 138 (14.6%).

Logistic regression analysis showed that duration of AF < 24 h (*p* < 0.001), left atrial volume index (LAVi) < 42 mL/m^2^ (*p* = 0.010), symptoms of near-collapse at presentation (*p* = 0.018), a lower BMI (*p* = 0.028), a longer QTc time during AF (OR 1.01, 95% CI 1.0–1.02, *p* = 0.002) and first-detected AF (OR 2.5, 95% CI 1.6–3.9, *p* < 0.001) were independent determinants of early SCV. Among those who experienced SCV those with a duration of AF < 24 h, first-detected AF, and smaller LAVI, the SCV rate was high: 38% (38 of 99 patients).

In 2021, Niederdöckl et al. [102] published an article describing “The ReSinus score”, a score easily used in the ED and able to predict SCV. This study analysed a retrospective cohort of 2426 cases of first-detected or recurrent, hemodynamically stable, non-permanent symptomatic AF in the Austrian Academic Emergency Department Atrial Fibrillation Registry between January 2011 and January 2019. In the derivation cohort, 1420 cases were included (68 years, 57–76; 43% female), while 1006 cases were included in the validation cohort (69 years, 58–76; 47% female). Multivariable analysis was used to develop and validate a prediction score for spontaneous conversion to sinus rhythm during ED visit. The clinical utility of the score was assessed using a decision curve analysis. Six variables that independently predicted SCV were found; each one of them was weighted in order to build the risk score. These included: the duration of AF-related symptoms (<24 h; 2 points), a lack of prior cardioversion history (2 points), heart rate at admission (>125 beats per minute; 1 point), potassium replacement at K + level ≤ 3.9 mmol/L (1 point), NTproBNP (<1300 pg/mL; 1 point) and lactate dehydrogenase level (<200 U/L; 1 point). The odds ratio associated with every increase of one score point was 1.61 (95% CI 1.47–1.76; *p* < 0.001). The risk score was then classified as of low (0–2), medium (3–5) and moderate (6–8) probability of spontaneous conversion. The rates of short-term spontaneous conversion to sinus rhythm across these three classes were 3.8%, 12.3%, 33.8% respectively. The final score showed good calibration (*p* = 0.44 and 0.40) and discrimination in both cohorts (indices: 0.74 and 0.67) and clinical net benefit. The fundamental aspect of this score is that its every component can be obtained in just one hour, making this tool suitable for real world practice. Spontaneous conversion to sinus rhythm occurred in 186 patients (13%). The observed incidence of short-term spontaneous conversion was 50% for patients with 8 points and 0% for patients with 0 points.

### 3.9. Different Percentage of SCV among Different Studies

**Figure 1:**
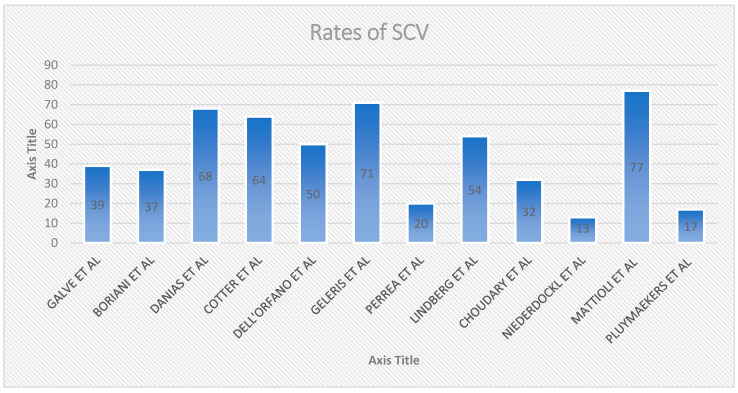
Different SCV% among studies.

If spontaneous cardioversion is well described in the context of paroxysmal atrial fibrillation (PAF), rates of SCV vary in different studies, accordingly to observation time and study population characteristics (Figure 1): Pluymaekers et al. (2021) [101] reports a SCV percentage of 16.8% in 3 h, Boriani et al. (1997) [89] 56% in 8 h, Choudary et al. (2013) [95], 46% in 18 h, Galve et al. (1996) 60% in 24 h [88], Cotter et al. (1999) 64% in 24 h [90], Geleris et al. (2001) 71% in 24 h [92], Mattioli et al. (2000) 77% in 48 h [99], Dell’Orfano et al. (1999), 77% in 48 h [94], Danias et al. (1998) 70% in 72 h [93]. Assuming population homogeneity, the main reason for the discrepancy between SCV rates lies in the different amount of time considered by the studies. Studies examining a longer ER observation time tended to report greater SVC rates. Study details can be found in Table 1.

## 4. Discussion

Living in the era of tremendous progress in medicine signifies that the general population nowadays has a much longer life expectancy. Furthermore, people lead very active lifestyles which they need to maintain. AF is the most common arrythmia worldwide and its incidence is not only rising but is expected to rise further as a consequence of the aforementioned prolonged life expectancy and the increasing prevalence of cardiovascular risk factors. Being very common, it represents a burden both to the healthcare system and patients as its consequences—for instance, HF or stroke—can lead to permanent disability, a declining quality of life or even death. To fight the growing burden of AF a deep knowledge of its causes and manifestations is required. In addition to a comprehensive understanding of the pathophysiology, a standardized and cost-efficient treatment is the most important weapon in the physician’s armamentarium in the fight against AF. Only when we understand all aspects of the patient with early onset AF—those being information regarding clinical, echocardiographic and laboratory findings—we can deliver the most appropriate treatment. Most symptomatic patients with AF will present to the ED, where a doctor will need to decide the correct approach to adopt in order to effectively treat the patient, in cost-effective and time-effective manner. For patients presenting with hemodynamic instability, ECV is, undoubtedly, the recommended treatment modality. However when patients are hemodynamically stable, a question arises: should they also undergo cardioversion? It is now known that early conversion to sinus rhythm is favorable, however a new aspect has arisen—several studies have reported the occurrence of spontaneous cardioversion without any medical intervention. A “watch-and-wait” strategy, reducing useless ECV and/or PCV would be welcomed, allowing early ED discharge and the reduction of AF-related economic costs for the healthcare structures. However, this approach should be tailored on patients characteristics, in order to choose the right treatment for the right patient in the right moment. As a result, specific patient characteristics which favor SCV should be sought in the ED to tailor each patient’s treatment.

A larger atrial size and declining left ventricular function are well known to play a major negative role in spontaneous conversion to SR as they may represent a tell-tale sign of atrial remodeling. When considering a “watch-and-wait” approach, an elevated atrial fibrillatory rate is very important, as well as the absence of previous episodes of AF. An exceedingly interesting predictor lies in the face of timing and the ever-growing research regarding human circadian rhythms, as it has been observed that AF with an onset during sleep has higher chances of undergoing SCV. Adequate potassium serum levels are fundamental too, as electrolyte abnormalities are known to prolong or even cause, cardiac arrythmias. NTproBNP and lactate dehydrogenase are equally important and easy to determine but are not the only laboratory parameter to be considered: a higher ANP value registered during the arrythmia has been shown to represent a favourable factor for SCV as it represents an indicator of correct atrial endocrine function—a feature lost with chronic AF.

Once identified, those predictors need to be collectively considered in a standardised manner in order to make a decision. Taking lessons from the simplicity of the CHADSVASC score and the efficiency of the APGAR, the possibility of using a Score, such as the “ReSinus score” may give ED doctors a weapon for taking decisions in a very short time, for a wide variety of patients. The physician can obtain all the information needed through medical history taking and rapid instrumental exams such as the 12-lead ECG and transthoracic echocardiography. In about 30 min every patient may be addressed to active cardioversion or after the prescription of adequate oral anticoagulation, dismissed.

## 5. Conclusions

In everyday practice in the ED, a clear protocol is needed to effectively manage diverse and often chaotic situations. Recognizing which patient would spontaneously cardiovert could help ED physicians to redirect time and resources to those who need an active approach to treat AF. The actual challenge is to define both the “ideal SCV patient” as well as the perfect amount of time for which a watch-and-wait strategy may be fruitful. The absence of HF, a small atrial size, recent-onset AF, a rapid AFR and the relationship between a past AF episode and HR/BP characterize patients who can just be treated with rate control and an early hospital discharge. In the era of precision medicine, such approach may help not only tailoring the treatment to the specific individual, but also reducing the ever-growing healthcare costs. 

## Figures and Tables

**Table 1:** Studies evaluating the rate and determinants of spontaneous cardioversion of atrial fibrillation.

Study, Year	**Study Design, Intervention**	**Setting, Observation Time**	**Patient Included**	**SCV Rate %**	**Main Determinant(s) of SCV**
Galve et al., 1996 [88]	IV Amiodaron VS Placabeo	ED/hospitalized, 24 h observation	Recent onset AF (<7 days)	24/62 (39%)	HF, Left atrial size
Boriani et al., 1997 [89]	Oral propafenone Vs Placebo	ED/Hospitalized, 8 h observation	Recent onset AF (<7 days)	45/121 (37%)	HF
Danias et al., 1998 [93]	Prospective	ED/hospitalized, observation 4.6 days	Recent onset AF < 72 h	242/356 (68%)	atrial fibrillation of 24 h duration at presentation
Cotter et al., 1999 [90]	IV Amiodaron VS Placabeo	ED/hospitalized, 24 h observation	Paroxysmal AF < 48 h and at least one previous episode of paroxysmal AF	32/50 (64%)	HF, left atrial size
Dell’orfano et al., 1999 [94]	Retrospective	ED < 48 h observation	Primary diagnosis of AF by 12 lead ECG or single channel	57/114 (50%)	Duration of AF
Mattioli et al. (2000) [99]	Prospective	ED 48 h observation	Consecutive with recent onset AF (<6 h)	108/140 (77%)	AF developed during sleep, ANP
Geleris et al., 2001 [92]	Prospective	ED 24 h observation	Consecutive patients with recents onset AF (<24 h)	109/153 (71%)	Left Atrial Size
Perrea et al., 2011 [97]	Retrospective study, SCV, Amiodaron	ED no observation time	AF at the time of presentation (<48 h)	28/141 (20%)	Heart rate, Blood Pressure, number of past episodes of AF
Lindberg et al., 2012 [91]	Retrospective	ED < 48 h observation	Consecutive patients admitted to hospital with first onset AF	203/374 (54%)	HF, Duration of AF
Choudary et al., 2013 [95]	Retrospective	ED SCV < 18 h after	Patients with Paroxysmal AF < 48 h	48/148 (32%)	Atrial Fibrillatory rate
Niederdöckl et al., 2020 [102]	Retrospective	ED atrial fibrillation confirmed by 12-lead electrocardiography.	First detected or recurrent hemodynamically stable non-permanent symptomatic atrial fibrillation	186/2426 (13%)	Resinus score (duration of af symptoms < 24 h, no previous electrical cv, heart rate > 125 bpm, potassium replacement at k+ level < 3.9 mmol/L, nt-probnp < 1300 pg/mL, ldh < 200 u/i
Pluymaekers et al., 2021 [101]	prospective	ED, 3 h	first detected hemodynamically stable non-permanent symptomatic AF	158/943 (16.8%)	duration of AF < 24 h, left atrial volume index (lavi) < 42 mL, symptoms of near-collapse at presentation, a lower BMI, a longer qtc time during AF, and first-detected AF

AF: Atrial fibrillation; ANP: Atrial Natriuretic Peptide; ECG: electrocardiogram; ED: Emergency Department; HF: Heart Failure; IV: Intra-venous; SCV: Spontaneous cardioversion.

## Data Availability

Not applicable.
